# The socio‐economic impact of running‐related injuries: A large prospective cohort study

**DOI:** 10.1111/sms.14016

**Published:** 2021-07-11

**Authors:** Tjerk S. O. Sleeswijk Visser, Marienke van Middelkoop, Tryntsje Fokkema, Robert‐Jan de Vos

**Affiliations:** ^1^ Department of Orthopedic Surgery and Sports Medicine Erasmus MC University Medical Center Rotterdam The Netherlands; ^2^ Leiden University Medical Center Leiden The Netherlands; ^3^ Department of General Practice Erasmus MC University Medical Center Rotterdam The Netherlands; ^4^ Department of General Practice and Elderly Care Medicine University Medical Centre Groningen University of Groningen Groningen The Netherlands

**Keywords:** activities of daily living, epidemiology, running, work

## Abstract

**Objectives:**

To evaluate the impact of running‐related injuries (RRIs) on activities of daily living (ADL), work, healthcare utilization, and estimated costs.

**Design:**

Prospective cohort study with data from a randomized controlled trial.

**Methods:**

Adult recreational runners who registered for a running event (distances 5 to 42 km) were included in this study. Minimum follow‐up duration was 3 months (preparation, event participation, and post‐race period). Injuries were registered using a standardized definition. Primary outcome measure was a standardized 5‐item survey on limitations in ADL. The survey data were categorized to the number of injured runners with complete/moderate/no limitations. This outcome was expressed as the percentage of injured runners with any limitation (complete or moderate limitations amalgamated). Secondary outcomes were work absenteeism, the number of healthcare visits per injured runner, and estimated direct medical and indirect costs per participant and per RRI.

**Results:**

1929 runners (mean [SD] age 41 [12] years, 53% men) were included in this study and 883 runners (46%) sustained a RRI during the course of the study. Injured runners reported the highest limitations (% with any limitation) of RRIs during the first week of injury on sports and leisure activities (70%) and transportation activities (23%). 39% of the injured runners visited a healthcare professional. Work absenteeism due to the RRI was reported in 5% of the injured runners. The total mean estimated costs were €74 per RRI and €35 per participant.

**Conclusions:**

Injured runners are mainly limited in their transportation activities and during sports and leisure. While the estimated costs of RRIs are not high when expressed per participant, the absolute costs may be substantial due to the popularity of running.

## 1 INTRODUCTION

Physical activity has proven to be a cost‐effective way to enhance overall health and reduce morbidity and mortality.^1^, ^2^, ^3^, ^4^ Running is an increasingly popular way to improve physical and mental well‐being.^5^, ^6^, ^7^ In 2019, close to 2 million people (11% of the Dutch population) performed weekly running activities in the Netherlands.^8^

Musculoskeletal injuries are a prominent disadvantage of running, with training errors being frequently suggested to be a major cause of injury.^9^, ^10^ Most running injuries are due to overuse and are located at the knee, lower leg, ankle, and foot.^11^, ^12^ The incidence of running‐related injuries (RRIs) varies among different populations (eg, cross‐country runners, novice runners, and long‐distance runners) of runners and can be up to 85% in novice runners training for an event.^9^ Even though RRIs are frequent, not much is known about the impact of these injuries on socio‐economic outcome measures. The impact of RRIs on activities of daily living (ADL) has, for example, never been described in literature.

Healthcare utilization, direct medical costs, and indirect costs due to absenteeism from work are outcome measures to estimate the impact of a disease.^13^ A few studies have reported the economic burden of RRIs, which varies between €83 and €174 per RRI and €13 and €105 per participant training for an event.^14^, ^15^, ^16^ These ranges are large and this may be due to the fact that these results were based on small study samples or only novice runners were included.^14^, ^15^ This makes it difficult to extrapolate these findings to the general recreational running population.^16^, ^17^ Therefore, it is relevant to evaluate the economic burden of RRIs in a large heterogeneous running population. Knowledge of the social impact, the specific areas affected by RRIs, and the experienced pain and disability could aid in the design of tailored treatment practices. The magnitude of the economic burden of RRIs is important to know, as it affects the urgency of RRIs in scientific agendas.

Therefore, the primary objective of this study is to assess the impact of RRIs on activities of daily living in runners training for an event. Secondary objectives are to evaluate the experienced pain and the effect of RRIs on work absenteeism, healthcare utilization, and estimated direct and indirect costs.

## 2 METHODS

### 2.1 Study design

The study was designed at the Erasmus MC University Medical Centre (Rotterdam, the Netherlands) and was part of a randomized control trial (The INSPIRE trial), which evaluated the effect of an online prevention program on the number of RRIs among recreational runners. A detailed study protocol has been published elsewhere.^18^ The Medical Ethics Committee of the Erasmus MC University Medical Centre Rotterdam, the Netherlands, approved the study protocol (MEC 2016–292). The trial was registered before commencement (NTR number: NL5843).

For the randomized trial, patients in the intervention group had access to an online injury prevention program, whereas the control group did not receive this information. There were no differences in injury proportion between both groups, and therefore, we regarded this study population as a large cohort. The results of this randomized controlled trial have been published elsewhere.^19^

In the RCT and the current study, an RRI was defined as an injury of the muscles, joints, tendons, and/or bones in the lower back or lower extremities that was caused by running with at least one of the following criteria: (1) the injury caused a reduction in running distance, speed, duration, or frequency for at least 1 week; (2) the injury led to a visit to a medical specialist and/or physiotherapist; and/or (3) medication was necessary to reduce symptoms as a result of the injury.

### 2.2 Participants

Potentially eligible participants were runners of 18 years or older who registered for one of 3 running events in 2017. These running events included the LadiesRun Rotterdam (5, 7.5 or 10 km), the NN Marathon Rotterdam (10.6 or 42.2 km), and the NN City Pier City The Hague (5, 10 or 21.1 km). If runners expressed their interest to participate during online registration for the event, they were provided with more information, and if still interested, they were assessed for eligibility. Participants were included if they met the inclusion criteria (18 years or older, registration at least 2 months before the running event, knowledge of the Dutch language, and access to email). After providing digital informed consent, participants could immediately complete the baseline survey.

### 2.3 Procedures

Patients were asked to complete an online survey (using the secure application LimeSurvey) on 4 different time points; (i) at baseline (≥2 months before the running event, (ii) 2 weeks before the running event, (iii) 1 day after the running event, and (iv) 1 month after the running event. At baseline, runners were asked to complete questions on demographics (sex, age, length, and weight), training characteristics during the past year (running frequency, duration, and speed), and lifestyle (smoking, alcohol use). The baseline survey also inquired whether the runner had suffered an RRI in the past 12 months. The 3 follow‐up survey consisted of questions about the current state of previously reported RRIs, the occurrence of new RRIs, the impact of new RRIs on ADL, and work absenteeism and health care utilization due to the RRI. Injured runners were asked to specify injury location (back, buttock, hip, groin, upper leg, knee, shin, calf, Achilles, ankle, foot, or toe) and injury onset (gradual or acute).

### 2.4 Outcome measures

#### 2.4.1 Primary outcome measure

Impact of RRIs on activities of daily living (ADL) was measured at all 3 follow‐up time points (2 weeks before the running event, 1 day after, and 1 month after the running event), using a 5‐item survey. Only participants who sustained one or multiple new RRIs were asked to complete this survey. Injured runners completed this survey only once per follow‐up time point, independently of the number of RRIs they sustained. This survey has not been validated but has been used in previous studies on RRIs.^20^, ^21^ The survey consists of 5 questions on the following dimensions: (1) daily activities (eg, getting up, washing, getting dressed), (2) household activities (eg, cleaning, vacuuming), (3) activities at work/school, (4) transportation activities (eg, driving, cycling, walking), and (5) sports and leisure activities. Each domain consists of 3 response options: no limitations, moderate limitations, and complete limitations. Injured runners were asked to indicate their ability to perform activities of daily living in the first week after the injury. For every follow‐up time point, injured runners completed this survey, resulting in an expression of the number (%) of injured runners with complete, moderate, or no limitations per domain. The results of these three separate follow‐up time points were combined and expressed as the total number (%) of injured runners with complete, moderate, or no limitations per domain. Results were also expressed as the number of injured runners with any limitation (complete or moderate limitations amalgamated). We also compared the impact on ADL in the first week after the injury between RRIs with an acute and gradual onset.

#### 2.4.2 Secondary outcome measures

##### Impact on ADL per RRI location

We compared the impact on ADL between different injury locations by subdividing the RRIs in 5 clustered injury locations: (1) lower back, (2) buttock/hip/groin, (3) upper leg/knee, (4) lower leg (shin/Achilles/ankle), and (5) foot/toe.^19^ If injured runners sustained more than 1 RRI, which originated from different clustered injury locations (eg, if a runner sustained an RRI to the groin and an RRI to the ankle), they were excluded from this part of the analysis. This is because in these cases, it was not possible to adequately assess which RRI specifically led to an impact on ADL. If injured runners sustained multiple RRIs, but these injuries were all located in the same clustered injury location, they were included in this part of the analysis. For every follow‐up time point, injured runners completed this survey, resulting in an expression of the number (%) of injured runners with any limitation (complete or moderate limitations amalgamated). Injured runners were asked to indicate the amount of pain (on a visual analogue scale; VAS 0–10) during rest and running in the week preceding the completion of the survey. We also compared the mean pain during rest and running between the clustered injury locations and between acute injuries and gradual onset RRIs.

##### Work absenteeism

Work absenteeism was assessed by the number of lost days at work/school due to an RRI and was measured at all 3 follow‐up time points. Only injured runners were asked to complete this part of the survey. Work absenteeism was expressed as the mean number of days of absence from work per injured runner.

##### Healthcare utilization

Injured runners were asked whether they had used health care due to an RRI. Healthcare utilization was assessed by asking the total number of healthcare visits per type of healthcare provider. Healthcare utilization was expressed as the mean healthcare consumption (number of visits) and mean medical costs per injured runner, per type of healthcare provider.

##### Costs

The estimated costs were divided into 2 categories: costs from healthcare utilization (direct costs) and costs as a result of absenteeism from work (indirect costs). We established productivity costs per hour and the costs per treatment/visit based on a guideline for economic evaluations in health care, published by the Dutch Healthcare Authority.^22^, ^23^ We determined the direct costs by multiplying the total number of visits/treatments with the estimated medical costs for those visits/treatments. The specific costs used for the economic evaluation are presented in Appendix S1. Mean direct and indirect costs due to an RRI were calculated per RRI and per participant (the mean of all participants and not only injured runners).

### 2.5 Statistical analysis

Presence of a normal distribution of data was assessed using the Shapiro‐Wilk test. Normally distributed data are presented as mean with standard deviation (SD) and non‐normally distributed data as median with interquartile range (IQR). We presented costs (in €) as mean with standard deviation (SD). Differences in direct, indirect, and total costs between men and women and between acute and gradual onset RRIs were compared using a Mann‐Whitney *U* test. Missing data (participants who did not complete at least one follow‐up survey) were excluded from the analyses for the study purposes described in this manuscript. For the analyses, a *p* value of <0.05 was considered statistically significant. We used SPSS software (V.24.0.0.1; SPSS) for statistical analysis.

## 3 RESULTS

### 3.1 Participants

In total, 2378 participants were included in the randomized control trial of whom 1929 participants (81%) completed at least one follow‐up survey and were included in this study. The mean (SD) age was 42^12^ years with the majority being male (53%). 883 (46%) participants reported at least one RRI during the course of this study. Most injuries (61%) had an acute origin. 714 of the 883 (81%) injured runners completed the surveys for our primary and secondary outcome measures. The participant characteristics are displayed in Table 1.

**TABLE 1:** Descriptive statistics of participants

Characteristics (*n* = 1929)	Mean (SD)
Personal characteristics	
Age (years)	41.9 (12.1)
Sex (Male/Female)	1020/909
BMI (kg/m^2^)	23.6 (2.9)
Injury‐related factors	
Injury proportion	883 (45.8%)
Injury mechanism (acute/gradual onset); %	61/39
Previous RRI (preceding 12 months) *n* (%)	994 (51.5%)
Reported RRI at baseline (yes)	415 (21.5%)
Sports‐related factors	
Running duration (hours/week)	3.1 (3.7)
Running experience (years)	6.8 (8.1)
Lifestyle‐related factors	
Smoking (yes) (%)	76 (3.9%)
Alcohol use (glasses per week)	4.2 (4.8)
Days with >30 mins of physical activity (days/week)	5.8 (2.0)

Note

Values are displayed in frequency means (standard deviation).

Abbreviations: BMI, body mass index; RRI, Running‐related injury; SD, standard deviation.

### 3.2 Primary outcome—Activities of daily living

Injured runners reported the highest limitations (any limitation) of RRIs during the first week of their injury on sports and leisure activities (70%) and transportation activities (23%). Lower frequencies of limitations were reported for daily activities (10%), household activities (12%), and activities at work/school (9%).

**FIGURE 1:**
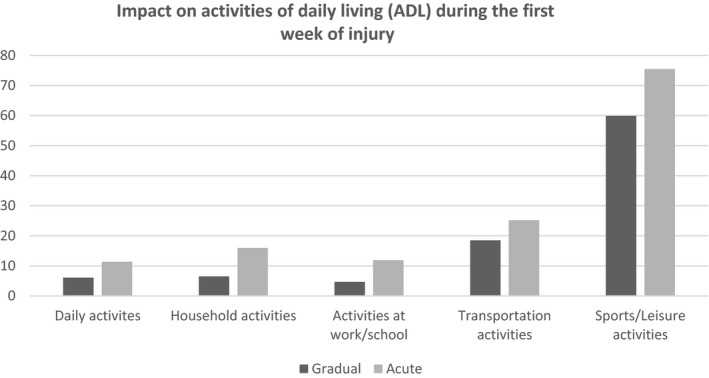
Impact on activities of daily living (ADL) during first week of injury (gradual vs. acute onset injuries). Displayed values are percentages of any (moderate and severe) limitation

Injured runners with acute onset RRIs reported higher limitations (any limitation) of RRIs during the first week of their injury compared to injured runners with gradual onset RRIs on daily activities (11% vs. 6%), household activities (16% vs. 7%), activities at work/school (12% % vs 5%), transportation activities (25% vs. 18%), and sports and leisure activities (75% vs 60%). Figure 1 shows the impact on ADL of acute and gradual onset RRIs.

### 3.3 Secondary outcomes

#### 3.3.1 Impact on ADL per RRI location

Injured runners with RRIs located at the lower back and lower leg reported higher limitations (any limitation) of RRIs during the first week of their injury compared to the overall average on household activities (42% and 13% vs. 12%), activities at work/school (25% and 10% vs 9%), transportation activities (42% and 26% vs. 23%), and sports and leisure activities (71% and 75% vs 67%).

**FIGURE 2:**
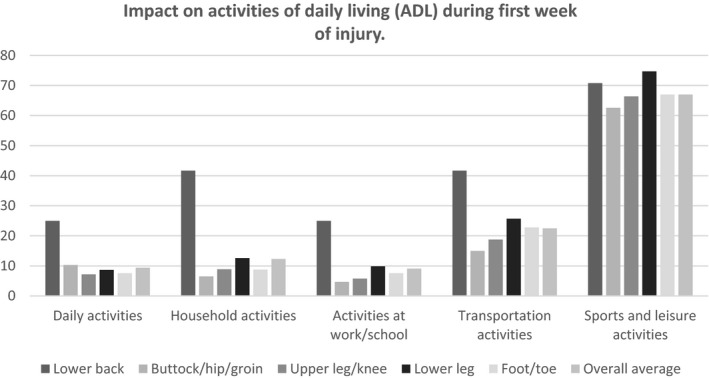
Impact on activities of daily living (ADL) during first week of injury (specified per injury location). Displayed values are percentages of any (moderate and severe) limitation

The impact on ADL per clustered injury location is shown in Figure 2. Mean (SD) pain (VAS 0–10) score during rest was higher in lower back (4.8 [2.8]) injuries compared to buttock/hip/groin (3.7 [2.4]), upper leg (3.5 [2.4]), lower leg (3.3 [2.9]), and foot injuries (3.2 [2.4]). Mean pain (VAS 0–10) score during running was lower in lower back injuries (5.1 [3.1]) compared to buttock/hip/groin (6.0 [2.7]), upper leg (5.9 [2.7]), lower leg (6.0 [2.9]), and foot injuries (6.4 [2.7]). Mean (SD) pain (VAS 0–10) scores during rest and running were similar for acute injuries (3.7 [2.4] and 5.7 [2.7]) and gradual onset RRIs (3.4 [2.4]) and 6.0 [3.0]).

### 3.4 Work absenteeism

Work absenteeism due to an RRI was reported in 5% of the injured runners. Within this group of injured runners, the mean (SD) number of days of absence from work due to an RRI was 3.5 (3.5).

### 3.5 Healthcare utilization

39% of the injured runners visited a healthcare professional and 8% initiated self‐care. The mean (SD) number of healthcare visits was 1.4 (4.5) per injured runner. A visit to a physiotherapist was reported by 32% of the injured runners. 4% visited a general practitioner and 2% reported having visited a medical specialist. 76% of the total number of healthcare visits consisted of physiotherapist visits. Table 2 demonstrates the frequencies of healthcare visits per type of healthcare provider.

**TABLE 2:** Healthcare utilization and medical costs per injured runner, per type of healthcare provider (*n* = 714 injured runners)

Healthcare provider	Patients using health care, no. (%)	Mean healthcare consumption (% of all healthcare visits)	Mean (SD) Medical costs
Primary care (visits)			
General practitioner	26 (3.6%)	0.06 (8.6%)	€2.11 (13.0)
Physical therapist	231 (32.4%)	1.2 (76.2%)	€39.88 (141.7)
Other^a^	31 (4.3%)	0.1(10.2%)	€3.25 (19.4)
Secondary care (visits)			
Medical specialist (eg, Sports medicine physician/orthopedic surgeon)	15 (2.1%)	0.04 (5.0%)	€3.48 (27.7)
Total		1.4 (100)	€48.74 (154.6)

Abbreviation: SD, standard deviation.

^a^ Another healthcare provider (eg, masseur, osteopath, podiatrist, alternative healthcare provider).

### 3.6 Estimated direct and indirect costs

The majority (82%) of the estimated total healthcare costs consisted of physiotherapy treatments (Table 2). The estimated total healthcare costs were €39 (SD 139) per RRI and €18 (SD 97) per participant, accounting for the entire study population of participants who completed at least one follow‐up survey (*N* = 1929). The estimated costs due to absenteeism from work were €35 (SD 267) per RRI and €16 (SD 183) per participant. Total estimated direct and indirect costs were €74 (SD 329) per RRI and €35 (SD 227) per participant (Table 3). Costs from work absenteeism accounted for 48% of the total costs.

**TABLE 3:** Direct and indirect costs per RRI (*n* = 901) and per participant (*n* = 1929)

	Overall	Mean (SD) medical costs	Mean (SD) indirect costs (absenteeism from paid work)
Cost per RRI, total (*n* = 898)	€74.29 (328.6)	€38.99 (138.5)	€35.29 (266.7)
Acute onset (*n* = 523)	€99.75 (420.6)	€40.74 (165.6)	€59.00 (346.6)
Gradual onset (*n* = 375)	€38.78 (100.0)	€36.56 (88.0)	€2.22 (32.1)
*p* Value	0.87	0.71	0.0
Cost per participant, total (*n* = 1929)	€34.58 (227.7)	€18.15 (96.9)	€16.43 (182.8)
Males (*n* = 1020)	€33.98 (225.7)	€20.35 (120.0)	€13.63 (169.2)
Females (*n* = 909)	€35.25 (230.3)	€15.68 (59.3)	€19.57 (196.9)
*p* Value	0.48	0.45	0.49

Note

Mann‐Whitney *U* test values comparing costs between acute and gradual onset RRIs and males and females.

Estimated direct costs for acute and gradual onset RRIs were €41 (SD 166) and €37 (SD 88), respectively. This difference was not statistically significant (U = 96 950.5, *Z* = −0.38, *p* = 0.71). The estimated indirect costs for acute RRIs (mean €59) were significantly higher (U = 92 953.0, *Z* = −4.2, *p* = 0.00) than for RRIs with a gradual onset (mean €2). There was no significant difference in total costs for acute RRIs (mean €100) compared to RRIs with a gradual onset (mean €39) (U = 97 563.0, *Z* = −0.17, *p* = 0.87).

No statistically significant difference in estimated direct and indirect costs was found between males and females (Table 3).

## 4 DISCUSSION

This study showed that the largest impact of RRIs is on sports and leisure (70%) and transportation activities (23%), while the impact on other activities of daily living was relatively low. The percentage of injured runners with any limitation in ADL was higher in RRIs located at the lower back and lower leg compared to the other clustered injury locations. Work absenteeism due to an RRI was reported in 5% of the injured runners. The total mean number of healthcare visits was 1.4 per injured runner, and the total mean estimated costs were €74 per RRI and €35 per participant. Acute injuries initially led to more limitations in ADL and higher estimated total costs.

### 4.1 Activities of daily living

Activities of daily living were mainly affected in the domains sports and leisure activities and transportation activities. Still approximately 1 in 10 injured runners experienced limitations in daily and household activities or activities in work/school. To better understand the impact of RRIs on activities of daily living, we evaluated this impact for both acute and gradual onset RRIs and for different injury locations. We found that acute onset RRIs and lower back and lower leg injuries in particular led more frequently to limitations in daily life. This limiting effect only partly correlated with pain score during rest, which was relatively high in lower back injuries. The restricting effect of lower back pain on ADL has been demonstrated in previous studies.^24^, ^25^ For lower leg injuries such as shin bone, Achilles tendon, and ankle joint injuries, this has never been described using this approach. Healthcare providers can take this into account when educating injured runners with lower back or lower leg injuries about the potential consequences of their injury. This information can also be used by healthcare policy makers in the design of tailored management plans or preventive measures.

### 4.2 Healthcare utilization, work absenteeism, and costs

Healthcare utilization mainly consisted of physiotherapy visits, which is in line with existing literature.^13^, ^14^, ^15^, ^16^ The total costs of RRIs were estimated at €35 per participant and €74 per RRI. We compared these costs to other studies describing the economic burden of RRIs in runners preparing for and participating in a running event. Two studies estimated the economic burden to be around €173 per RRI.^14^, ^15^ In contrast to our study, only small and selected (trailrunners and runners participating in events ≤10 miles) running populations were included. This could have led to a less accurate estimation of the economic burden of RRIs in these studies and less generalizability to the overall running population. A large prospective cohort study estimated the cost of RRIs to be €13 per participant.^16^ However, this study had a follow‐up of only 6 weeks. This could explain why the estimated costs in the current study are higher as long‐standing injuries will have had more impact on our study.^16^, ^26^, ^27^ Expressed per participant the total costs of RRIs appear to be relatively low. When considering the popularity of running, which is practiced by close to 2 million people in the Netherlands, the absolute costs of RRIs may be substantial. This underlines the need for optimized preventive measures.

Previous studies reported contradictory findings on the difference in costs between males and females and between acute and gradual onset RRIs.^14^, ^15^, ^16^ The costs per RRI in the current study were substantially higher for injuries with an acute onset than injuries with a gradual beginning. This difference can be explained by the difference in costs from absenteeism from work. The finding of higher indirect costs for acute RRIs is in line with a large study on RRIs among novice runners but in contrast with other studies.^14^, ^15^, ^16^ Overuse injuries are supposed to have more impact over time, which was demonstrated in several studies.^16^, ^26^, ^27^ If we would have followed the runners for a longer time, costs of overuse injuries may have increased. It could be hypothesized that RRIs with an acute onset are accompanied with higher absenteeism from work because the severity of symptoms is higher in the initial phase of the injury, while the severity of injuries with a gradual onset is spread out over time. This might lead to less people being absent from work. We found that injured runners with acute onset RRIs experience more limitations on ADL in the first week of injury, which could support this hypothesis. The difference between both injury groups could also originate from the way we measured indirect costs in this study, as we only included costs from absenteeism from work and did not ask participants about a decrease in work productivity. A decrease in work productivity could lead to substantial costs, which has been shown in several studies on the impact of overuse injuries.^28^, ^29^, ^30^ It could be that—while acute onset injuries lead to higher absenteeism in the short term—once back at work the work productivity in people who suffered this type of injury is back to normal. Subsequently, indirect costs could be similar in both injury groups if they were measured over a longer period of time and measured more accurately (including work productivity measures).

### 4.3 Strengths and limitations

This is the first study to report the impact of RRIs on ADL. We were able to show this impact for different injury locations. Furthermore, this prospective cohort study included a large heterogeneous running population, which increases the generalizability of these findings. There are also some limitations to this study. We assessed our primary outcome by using a non‐validated survey, which could decrease the reliability of the results. However, we used an online survey with limited response options, which was completed in the same way by all injured runners at all 3 follow‐up time points. This guaranteed the internal consistency of the survey, and it is therefore likely that these results are reliable. Secondly, we asked patients about their limitations in daily life during the first week of injury retrospectively. This could have induced recall bias and may have led to inaccuracy of the results on this specific outcome measure.

### 4.4 Recommendations for future research

Future research could focus on the impact of RRIs on quality of life, hereby using validated questionnaires (eg, the EuroQol questionnaire [EQ‐5D]). This will provide more information on the social impact of RRIs and the specific domains which are affected. In addition, it would be interesting to perform an economic evaluation of RRIs with the addition of work productivity and a longer follow‐up period in order to evaluate if this affects the direct and indirect costs of overuse injuries. Next to this, it would be helpful if more economic evaluations per type of sports are performed to be able to adequately compare the costs of RRIs with different sports.

## 5 PERSPECTIVE

This study showed that runners suffering from an RRI are mainly limited in their sports and leisure and transportation activities and these limitations are particularly substantial in lower back and lower leg injuries. The total costs for runners training for an event were €74 per RRI and €35 per participant. Even though the estimated costs of RRIs are not high when expressed per participant, the absolute costs may be substantial due to the popularity of running and because long‐standing RRIs may further increase the costs with longer follow‐up time. Consequently, this study emphasizes the need for optimized preventive measures.

## CONFLICT OF INTEREST

The authors declare there is no conflict of interest.

## Supporting information

Appendix S1

## Data Availability

The authors can confirm that all relevant data are included in the article and/or its Supplementary Information Files.
