# Supplementary material for: The socio‐economic impact of running‐related injuries: A large prospective cohort study
Source: Scand J Med Sci Sports. 2021 Jul 11;31(10):2002–9. doi: 10.1111/sms.14016 (PMC8518541; doi:10.1111/sms.14016)
Supplement: Supplementary file 1 — Appendix S1 [file SMS-31-2002-s001.docx]

**Supplementary File 1. Cost used in the economic evaluation.**

| **Direct healthcare costs per visit** | **Euro’s** |
| --- | --- |
| General practitioner (<20 min) | 33.0 |
| Sports Physician/Orthopedic Surgeon | 91.0 |
| Physiotherapist | 33.0 |
|  |  |
| **Indirect costs** |  |
| Absenteeism from paid work (costs/hour) | 34.75 |

**Table 1. Costs used for the economic evaluation**
